# Evaluating the impact of interactive and entertaining educational conferences

**DOI:** 10.1007/s40037-013-0074-z

**Published:** 2013-08-07

**Authors:** Karen Jerardi, Lauren Solan, Dominick DeBlasio, Jennifer O’Toole, Christine White, Connie Yau, Heidi Sucharew, Melissa D. Klein

**Affiliations:** 1 Division of Hospital Medicine, Cincinnati Children’s Hospital Medical Center, 3244 Burnet Ave, 5th Floor, Kasota Bldg., MLC 9016, 45229-3039 Cincinnati, OH USA; 2 Division of General and Community Pediatrics, Cincinnati Children’s Hospital Medical Center, Cincinnati, OH USA; 3 Health Management and Policy, School of Public Health, University of Michigan, Cincinnati, OH USA; 4 Division of Biostatistics and Epidemiology, Cincinnati Children’s Hospital Medical Center, Cincinnati, OH USA

**Keywords:** Morning report, Medical education, Pediatrics, Resident conference

## Abstract

Adult learning theory suggests that meaningful engagement impacts learning. To evaluate the impact of resident-led interactive conferences on satisfaction, engagement and knowledge acquisition. A prospective study at a paediatric academic institution compared educational conferences in two formats. Control conferences were PowerPoint^®^ lectures and intervention conferences included multimedia, audience participation and faculty mentorship. Learner and presenter satisfaction and learner engagement were assessed by post-conference surveys. Knowledge was assessed via pre- and post-conference open-ended questions; matched pre- and post-questions were analyzed. Control and intervention groups’ satisfaction and engagement were compared using the Wilcoxon rank-sum test. Comparison of proportion of learners with improved post-conference knowledge score was analyzed with the Chi square test. There were 181 control and 170 intervention surveys collected. Learners’ median satisfaction (4 vs. 5, *p* = .03) and engagement (4 vs. 5, *p* < .01) ratings improved in the intervention group. Presenters rated audience engagement higher during the intervention conferences (median 3 vs. 4, *p* = .01). Knowledge acquisition, compared for matched surveys only, was not significantly different between the groups. Learner and presenter satisfaction and learner engagement were higher for the interactive format. While knowledge acquisition was unchanged, greater satisfaction encourages the use of interactive conferences.

## Introduction

Medical conferences are traditionally taught in a didactic fashion utilizing methods that encourage passive learning. Adult learning principles, pioneered by Malcolm Knowles, promote making knowledge relevant to the learner, building on learners’ prior experiences and creating an active learner-centred environment [1, 2]. Research has suggested that the use of adult learning theory in educational conferences can improve knowledge retention by having engaged and activated learners [3].

United States residency programmes are required to provide protected time for education [4]. However, residents and programme directors have expressed recent concern regarding the potentially negative effects of duty hour regulations on education [5, 6]. Therefore, it is imperative that conferences are effective and efficient. While learning during direct patient care remains a key method of resident education, conferences are another source of education that complements the clinical experience.

Current ‘Millennial’ generation learners are highly technologically literate and tend to prefer visual, interactive and experiential learning over reading and didactic lectures [7–9]. Previous work in this area found that an overwhelming majority of American medical students have a positive attitude about the use of technology to enhance their education and believe that technology could be used more often and effectively in their education [10]. Furthermore, recent literature has proposed that increased learner enjoyment and entertainment may increase meaningful learning [9, 11].

Our study compared the impact of morning report conferences utilizing multimedia technology in an entertaining and engaging format (intervention) to morning report conferences with a traditional didactic format (control) on knowledge acquisition, learner engagement, and learner and teacher satisfaction.

## Methods

This was a non-randomized study of an educational intervention conducted from November 2011 to February 2012. It was approved as exempt by the Cincinnati Children’s Hospital Medical Center (CCHMC) Institutional Review Board. CCHMC is a free-standing children’s hospital and paediatric residency training programme with over 180 residents and 150 rotating third-year medical students annually. All residents and medical students were invited to participate via email and received a copy of the consent form prior to the study’s initiation; however, no participants or presenters were aware of the aims or hypotheses of the study.

A total of 10 morning report conferences, all 30 min in length, during the four-month study period were randomly assigned to control or intervention format in a 1:1 ratio. Common inpatient and outpatient general paediatric topics were selected by the study team. In the control conferences, residents developed and presented a standard PowerPoint^®^ lecture. For the intervention conferences, residents developed and presented an interactive morning report utilizing at least one activity from a list of options encouraging audience participation (audience response systems, skits, games) and entertainment (music, rhymes/memory aids, podcasts). The intervention teams were mentored by an assigned faculty member with medical education training who ensured the interactive elements were incorporated.

Medical student and resident knowledge acquisition was compared between control and intervention conferences. Pre- and post-conference knowledge tests consisting of three open-ended questions were graded as correct or incorrect by a single-blinded faculty member.

The learners’ survey included three 5-point Likert scale questions to determine participants’ attitudes about the quality of knowledge gained, satisfaction with the educational format and self-reported engagement. Presenters were asked to rate audience engagement on a 5-point Likert scale and document the amount of time required to prepare for the conference.

Pre- and post-test forms were matched by a unique identifier. Attitude surveys were completed anonymously by both the learners and presenters and collected and entered into a secured database by a designated research assistant.

Knowledge acquisition was dichotomized as improved (post-conference knowledge question scores were higher than pre-conference scores) or not improved (post-conference knowledge scores were the same or lower). The difference in percentage of learners with improved scores between the two groups was compared using a Chi square test. A stratified analysis for each level of learner (medical student, intern and senior resident) was also conducted.

Differences in post-conference attitude survey responses from audience and presenters between the intervention and control groups were compared using Wilcoxon rank-sum test and a stratified analysis was conducted for each level of learner. The time presenters spent preparing for this report relative to past reports was dichotomized as ‘more time’ or ‘not more time’ (‘same’ or ‘less time’) and was compared using Chi square test.

## Results

A total of 144 matched pre- to post-conference knowledge tests were collected in the control group and 125 in the intervention group. Knowledge acquisition between the two formats was not significantly different: 56 % of learners in the control and 60 % of learners in the intervention group demonstrated improved post-conference (*p* = .53). Also, there were no differences in knowledge gain between conference formats for the three groups of learners: medical students, interns and senior residents. Learners reported significantly higher levels of satisfaction and engagement during the intervention conferences compared with the control conferences (Table 1).

**Table 1:** Summary of learners’ attitude survey questions

	Quality of knowledge			Satisfaction with educational format			Engagement		
	Control	Intervention	*p*	Control	Intervention	*p*	Control	Intervention	*p*
All learners									
*n*	180	169		181	170		181	170	
Median	4	4	.22	4	5	.03	4	5	<01
Medical students									
*n*	35	34		35	34		35	34	
Median	4	5	.02	5	5	.38	4	5	.20
Interns									
*n*	79	79		80	79		80	79	
Median	4	4	.36	4	5	.03	4	5	<.01
Senior Residents									
*n*	66	56		66	57		66	57	
Median	4	4	.39	4	5	.55	4	5	.01

Data presented as median for Likert scale scores ranging from 1 = lowest to 5 = highest. The 25th and 75th percentile was 4 and 5 for all questions

*p* values for difference between intervention and control groups from Wilcoxon rank sum test

All presenters completed the attitude survey (control conferences *n* = 20 and intervention conferences *n* = 21). Presenters reported feeling their audience was more engaged during the intervention conferences with a median rating of 4 (IQR: 4, 4.5) compared with the control conferences with a median rating of 3 (IQR: 3, 4), (*p* = .01). Additionally, there was no significant difference in the percentage of presenters reporting more time preparing for the conference in this study relative to preparation for past conferences between intervention (14 %) and control (22 %) groups (*p* = .57).

Resident presenters in the intervention group creatively approached structuring their conference including videos, songs, dancing and games. Examples included a relay race to identify appropriate isolation precaution attire for a clinical case, use of smart phone audience response systems for teaching developmental milestones, and artistic demonstration of physical exam findings pathognomonic for causes of pharyngitis.

## Discussion

Utilizing interactive and engaging educational techniques can enhance knowledge retention. These types of educational experiences are particularly critical for ‘Millennial’ learners who seek interactive and experiential learning opportunities [9]. This study achieved success by encouraging learners to use interactive and engaging methods of instruction during conferences at our institution. During our study, learners at multiple levels of training (medical students, interns and senior residents) all rated their engagement higher during the intervention conferences.

Adult learning theory suggests that meaningful engagement plays an important role in learning [2]. A qualitative study of medical education in the primary care setting proposes that the highest quality learning occurs for learners who are both engaged and exposed to adequate clinical opportunities [12]. Furthermore, the hallmarks of deep learning mirror those of adult learning theory; deep learning is motivated by the learner’s interest in the material and their ability to apply the material in real use. Deep learning stands in contrast to superficial learning that occurs during rote memorization without application [13]. By more effectively engaging our learners via interactive and engaging conference, we aim to foster deep learning by encouraging retention of knowledge and application to clinical scenarios.

As the number of learners from the ‘Millennial’ generation has increased, so has the use of technology in medical education. Indeed, a recent survey of medical students and residents showed that 95 % agreed that ‘smartphones will increase in usage in the future of medical education’ [14]. Technology will likely help keep medical conferences learner-centred and consistent with their generational paradigm. We, therefore, posit that the inclusion of multimedia technology played a significant role in increasing learner engagement [7, 8].

Resident presenters also rated audience engagement higher during the intervention reports. While this is presenter opinion, there is likely an advantage to presenter satisfaction and desire to teach when they feel their audience is more engaged. While we found higher learner engagement and satisfaction in the intervention conferences, we also found that the knowledge acquisition did not differ significantly. Learners gained knowledge in both conference formats. Since the literature supports that engagement and enjoyment lead to deeper learning, it is possible that there was an improvement in knowledge acquisition that we were unable to measure. This lack of detection of difference in knowledge acquisition may have been due to our limited three-question knowledge test; a more extensive knowledge test may have shown a measurable improvement. Additionally, the interactive elements of the presentations may have distracted learners from factual content potentially impacting knowledge gain.

We acknowledge that this study has limitations. This study occurred at a single site with learners from one residency programme and medical school. Further, this study looked at immediate change in knowledge and did not address knowledge retention or application. Presenter’s ratings of audience engagement may have been biased by their instruction to include interactive teaching methods.

## Conclusions

Interactive, engaging and entertaining resident-led conferences resulted in increased learner and presenter engagement while maintaining similar preparation times. While change in knowledge acquisition was not detected on the limited three question test, learner satisfaction did improve. Since engaged and satisfied learners are more likely to experience deep, meaningful learning, we believe that by utilizing technology and applying adult learning theory to optimize resident educational experiences, we may be able to enhance the effectiveness of our educational efforts.

## Abbreviations

ACGME: Accreditation Council for Graduate Medical Education
AAP: American Academy of Pediatrics
